# Effects of the Alkylating Agent Cyclophosphamide in Potentiating Anti-Tumor Immunity

**DOI:** 10.3390/ijms26136440

**Published:** 2025-07-04

**Authors:** Benjamin D. Gephart, Don W. Coulter, Joyce C. Solheim

**Affiliations:** 1 Eppley Institute for Research in Cancer and Allied Diseases, University of Nebraska Medical Center, Omaha, NE 68198, USA; 2 Fred & Pamela Buffett Cancer Center, University of Nebraska Medical Center, Omaha, NE 68198, USA; 3 Department of Pediatrics, University of Nebraska Medical Center, Omaha, NE 68198, USA; 4 Children’s Nebraska, Omaha, NE 68198, USA; 5 Department of Biochemistry & Molecular Biology, University of Nebraska Medical Center, Omaha, NE 68198, USA; 6 Department of Pathology, Microbiology & Immunology, University of Nebraska Medical Center, Omaha, NE 68198, USA

**Keywords:** cancer, chemotherapy, cyclophosphamide, immune, immunomodulation

## Abstract

Cyclophosphamide (CPX) is an alkylating agent commonly used for various hematological and solid malignancies. In addition to its use as a cytotoxic agent to directly kill tumor cells, numerous immunomodulatory properties of CPX in the tumor microenvironment (TME) of several cancer types have also been documented. These properties include the selective depletion of immune-suppressive regulatory T cells (Tregs), triggering of immunogenic cell death (ICD) and enhanced antigen presentation, and release of type I interferons (IFNs). Moreover, preclinical models as well as human clinical trials have investigated the efficacy of the low-dose “metronomic” scheduling of CPX in combination with immunotherapies such as immune checkpoint inhibitors, dendritic cell tumor vaccines, and tumor antigen peptide vaccines. The metronomic dosing schedule involves administering a continuous (or frequent, such as daily) low dose of chemotherapy rather than using the canonical approach of administering the maximum tolerated dose. Despite the approval of immune checkpoint inhibitors for clinical usage against an increasing number of cancers, many malignancies simply do not respond to checkpoint inhibition, in part due to the heterogeneous intratumoral network of immune-suppressive cell populations. The immunomodulatory effects of cyclophosphamide have strong translational applicability and could serve to enhance and bolster anti-tumor immunity, potentially synergizing with immune checkpoint inhibitors and other existing immunotherapy agents.

## 1. Introduction

Chemotherapeutic agents have historically been a part of the standard treatment modalities for many malignancies, and they serve to directly destroy actively dividing tumor cells. Despite the use of chemotherapies as the backbone of anti-cancer treatment regimens, many patients eventually develop therapeutic resistance and tumor relapse. While the development of immune checkpoint inhibitors and other novel immunotherapies for use as second-line therapies has been a significant advancement in the treatment of cancer, unfortunately many patients do not achieve complete remission and therefore still succumb to their disease.

There are several tumor types (so-called “cold” tumors) that tend to lack an inflammatory phenotype and the presence of anti-tumor infiltrating immune cells [1], which is partially attributable to the highly immune-suppressive tumor microenvironment (TME) in these cancers. Tumors can contain myriad immune-suppressive cell populations such as M2 macrophages, regulatory T cells (Tregs), and myeloid-derived suppressor cells (MDSCs) that release cytokines and growth factors which lead to impaired anti-tumor immunity. Moreover, these cell populations have been well known to express high levels of cell-surface immune-inhibitory molecules such as cytotoxic T-lymphocyte-associated protein 4 (CTLA-4), programmed cell death protein 1 (PD-1), and programmed death-ligand 1 (PD-L1).

**Figure 1:**
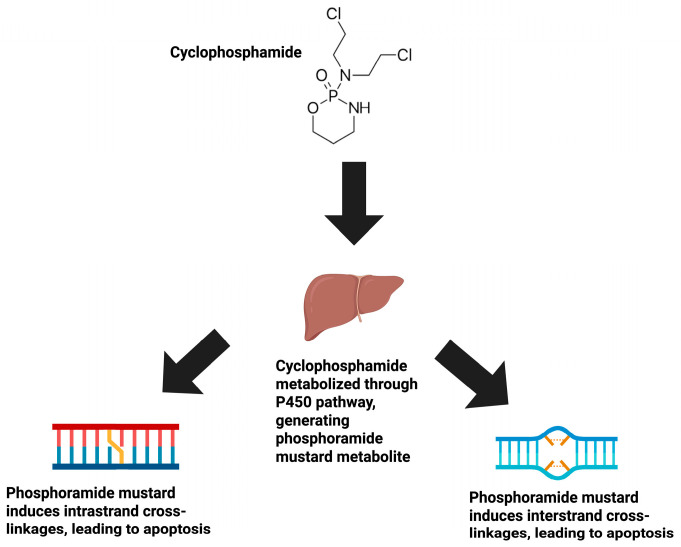
The mechanism of action of CPX. CPX exists as a prodrug compound, requiring subsequent biochemical metabolism via the P450 pathway in the liver. This biochemical pathway converts the CPX prodrug into its active metabolite, phosphoramide mustard. Phosphoramide mustard then proceeds to induce intrastrand and interstrand cross-linkages at the N7 position of DNA in actively dividing tumor cells, leading to apoptosis.

Cyclophosphamide (CPX) has been routinely utilized as a chemotherapeutic for a variety of malignancies, including lymphoma, leukemia, and neuroblastoma. In vivo, CPX is metabolized by the liver via the cytochrome P450 pathway, resulting in multiple metabolites (Figure 1). The primary CPX metabolite that initiates the cytotoxic effect of this drug is phosphoramide mustard. Phosphoramide mustard forms irreversible cross-linkages in and between deoxyribonucleic acid (DNA) of actively dividing tumor cells at the N-7 guanosine position, leading to cell death [2].

**Figure 2:**
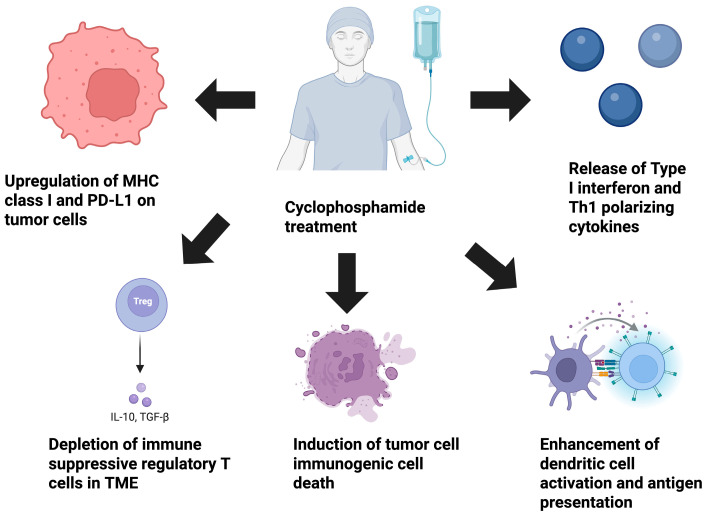
The immunomodulatory properties of CPX. The schematic image articulates the immune-modulating properties of the alkylating agent CPX. Preclinical studies of CPX have demonstrated that it is capable of restructuring the TME and bolstering anti-tumor immunity. CPX can deplete immune-suppressive regulatory T cells, trigger ICD through damage-associated molecular pattern release, augment dendritic cell antigen presentation, and modify the cytokine milieu within the TME through promoting the release of type I IFN and Th_1_ polarizing cytokines such as IFN-γ and IL-12. Lastly, CPX can enhance the immunogenicity of tumor cells directly by up-regulating MHC class I expression (enhancing the ability of cytotoxic T cells to eliminate tumor cells), and the immune checkpoint molecule PD-L1 (increasing susceptibility to PD-L1 checkpoint blockade). CPX, cyclophosphamide; ICD, immunogenic cell death; IFN, interferon; TME, tumor microenvironment; MHC, major histocompatibility complex class I; PD-L1, programmed death-ligand 1.

In recent years, there has been considerable interest in the immunomodulatory properties of chemotherapeutic agents, including the immunomodulatory effects of CPX. In this review, the immunomodulatory properties of CPX will be outlined, specifically its abilities to selectively target immune-suppressive Tregs, promote the induction of immunogenic cell death (ICD), improve antigen presentation, affect immune checkpoint and major histocompatibility complex (MHC) class I expression on tumor cells, and induce type I interferons (IFNs) (Figure 2). Due to the discovery of these effects, CPX has been identified as a means to induce an immunologically “hot” tumor phenotype in immunologically “cold” tumor types. This review will also present the potential advantages of using a metronomic dosing schedule of CPX concurrently to bolster the impact of immunotherapies such as immune checkpoint inhibitors, tumor antigen vaccines, and dendritic cell vaccines.

## 2. Selective Depletion of Immune-Suppressive Cell Populations Within the TME

Studies have documented the capability of CPX to selectively target and deplete immune-suppressive cell populations such as Tregs. Under normal physiological conditions, Tregs serve to maintain peripheral tolerance and restrain autoimmunity [3]. In the context of the TME, Tregs are highly effective at dampening anti-tumor immunity through several mechanisms, including the following: the expression of constitutively high levels of the immune checkpoint molecules CTLA-4 and PD-1, release of immune suppressive cytokines such as interleukin (IL)-10 and transforming growth factor (TGF)-β, metabolic consumption of IL-2 through expression of the high affinity chain of the IL-2R (CD25), and direct killing of effector CD8^+^ T cells [4].

Multiple studies have highlighted the ability of CPX to selectively deplete immune-suppressive Tregs in the TME. While the exact mechanism by which CPX selectively targets Tregs is still not fully understood, in vitro studies have suggested that Tregs express low levels of the adenosine triphosphate (ATP) influx and efflux receptors CD39 and CD73. Both CD39 and CD73 are important mediators of intracellular ATP levels. ATP, while commonly known as an energy source, is also integral for the generation of the antioxidant glutathione. Glutathione has been shown to reverse the DNA damage associated with CPX metabolites. The low expression of Treg CD39/73 reduces Tregs’ ability to correct and repair CPX-mediated DNA damage, leading to cell death [5]. A study published by Leong et al. in 2019 demonstrated that low-dose CPX (at 40 mg/kg) significantly reduced the frequency of intratumoral Tregs in a syngeneic CT26 murine model of colon carcinoma [6]. The investigators further reported that low-dose CPX synergized with a novel in situ vaccination with a CpG-class Toll-like receptor (TLR9) agonist, generating superior induction of CD8^+^ T cell responses and polarization of macrophages towards an M1 phenotype. Moreover, a report published by Zhong et al. demonstrated that treatment of a murine model of Lewis lung carcinoma with 150 mg/kg CPX reduced CD4^+^CD25^+^FoxP3^+^ Tregs (in both the spleen and TME) and increased the proportion of CD4^+^ and CD8^+^ T cells [7]. Huang et al. published data further demonstrating that low-dose metronomic CPX administration significantly reduced levels of intratumoral Tregs and reduced the levels of IL-10 and TGF-β in a metastatic murine model of CT26 colon adenocarcinoma [8]. In short, several preclinical models of cancer have shown the potency of CPX in selectively depleting Tregs in the TME, which serves to shift the ratio of effector CD8^+^ T cells to Tregs toward a greater proportion of effector CD8^+^ T cells in the TME. This ratio of CD8^+^ T cells to Tregs has been well characterized as a crucial positive prognostic indicator in multiple cancer types. Thus, shifting the balance of T cell populations to favor greater quantities of CD8^+^ T cells by selectively depleting Tregs with chemotherapeutic agents such as CPX is a strategic tactic that warrants significant interest.

## 3. Influence of CPX on ICD in Cancer

ICD is an inflammatory and immunogenic form of apoptosis whereby damaged tumor cells release damage-associated molecular patterns that can be taken up by antigen-presenting cells such as dendritic cells and lead to the induction of anti-tumor immunity. These damage-associated molecular patterns include high-mobility group box protein 1 (HMGB1), ecto-calreticulin (ecto-CRT), and extracellular ATP [9,10]. HMGB1 is a chromatin-binding protein that plays a role in regulating transcription by interacting with chromatin, nucleosomes, and histones. CRT is a chaperone protein that is normally expressed on the endoplasmic reticulum and sarcoplasmic reticulum of eukaryotic cells. During states of cellular stress, both HMGB1 and CRT are translocated to the cell surface. HMGB1 is released into the extracellular space while CRT is directly expressed on the cell surface. Several in vivo and in vitro studies have indicated that CPX is able to induce ICD. A study published by Webb et al. in 2022 showed that low-dose CPX induced HMGB1 release, using both in vitro and in vivo models of neuroblastoma [11]. The induction of ICD has been shown to increase dendritic cell activation and lead to more robust T helper 1 (Th_1_) immunity and superior priming of CD8^+^ T cell responses. The release of HMGB1 has been demonstrated to increase dendritic cell activation and antigen presentation capability through the activation of TLR2 and TLR4 on dendritic cells [12]. CRT expression of damaged tumor cells has been shown to also enhance dendritic cell antigen presentation and IL-12 secretion through the activation of the TLR4/MyD88 signaling pathway.

## 4. CPX’s Impact on Myeloid Cell Activation

Myeloid cells, such as macrophages and dendritic cells, are antigen-presenting cells that are key mediators of robust anti-tumor immunity [13]. Macrophages can also phagocytose and directly kill tumor cells through the release of lytic enzymes and reactive oxygen species. Dendritic cells are professional antigen-presenting cells that are regarded as potent activators of CD4^+^ and CD8^+^ T cell responses. Investigators have shown that low-dose CPX can trigger ICD, which has been shown to enhance antigen presentation and lead to T cell responses in various in vivo models of cancer [14,15]. Low-dose CPX has been shown to restructure the TME, polarizing macrophages to an M1 anti-tumoral phenotype and increasing the production of IL-6 and IL-12 [16]. Moreover, such M1 macrophages generated reduced levels of IL-10 and TGF-β. The triggering of ICD due to low-dose CPX treatment has been shown to produce abundant damage-associated molecular patterns that are potent ligands of dendritic cell TLRs, and their binding to these TLRs induces dendritic cell maturation and antigen presentation. Specifically, low-dose CPX has been shown to release damage-associated molecular patterns such as extracellular ATP and Annexin A1 in multiple in vitro studies, leading to the increased dendritic cell activation of T cell responses [17].

Using a model of colon carcinoma, Radojcic et al. published in 2010 a report that showed low- to moderate-dose CPX was capable of triggering increased production of IL-12 and tumor necrosis factor (TNF)-α, key indicators of Th_1_ immunity [18]. Shurin et al. (2009) further articulated the effects of low-dose CPX on dendritic cell activation, showing significant up-regulation of the co-stimulatory molecules and antigen-presenting molecules CD40, CD80, CD86, and MHC class II [19]. One of the key bottlenecks in effective cancer immunotherapy responses is proper antigen presentation. The immune suppressive nature of the cancer TME can often render antigen-presenting cells such as dendritic cells unable to properly activate effector immune cell populations. Consequently, these studies highlight a novel application of low-dose CPX to improve defective antigen presentation.

## 5. Direct Effects of CPX on Tumor Cells

Previous studies in preclinical models of cancer have indicated that chemotherapeutic agents such as CPX can often have direct effects on tumor cells that are relevant to immune responses, including up-regulating the expression of immune checkpoint molecules such as PD-L1 and of MHC class I molecules [6,20,21,22,23]. The tumor cell up-regulation of immune checkpoint molecules is a critical immune escape mechanism, and this provides the scientific basis for the use of immune checkpoint inhibitors for multiple tumor types. MHC class I is a critical antigen-presenting molecule that is required for direct, antigen-specific tumor cell killing by CD8^+^ T cells. As an additional immune escape mechanism, tumor cells will often down-regulate MHC class I expression to evade detection and lysis by CD8^+^ T cells. In a study published by Peng et al. in 2015, low doses of CPX were shown to up-regulate surface PD-L1 and MHC class I expression in various in vitro experiments using ID8 ovarian cancer cells [24]. Low-dose CPX further sensitized ID8 tumor-bearing mice to anti-PD-L1 blockade and increased the proportion of effector tumor-infiltrating leukocytes in vivo. Moreover, in a study published by Khan et al. in 2020, low-dose CPX was once again shown to potentiate up-regulation of PD-L1, sensitizing 4T1 triple-negative breast cancer tumor-bearing mice to anti-PD-L1 blockade in vivo [20]. These studies highlight a novel treatment paradigm in which low-dose chemotherapy can be used strategically to up-regulate key immune checkpoint molecules to enhance the clinical efficacy of immune checkpoint blockade in immunologically cold tumor types.

## 6. CPX Stimulation of Type I IFN Release

Type I IFNs comprise a subset of cytokines that modulate and control inflammation and the activity of various immune cell types such as T cells, B cells, and dendritic cells. Type I IFN has been shown to play a role in both anti-viral and anti-tumor immunity. The activation of the receptors for IFN-a and -b induces downstream signaling via Janus kinase/signal transducers and activators of transcription (JAK/STAT), mitogen-activated protein kinase (MAPK), nuclear factor kappa B (NF-κB), and phosphoinositide 3-kinase/Akt (PI3K/Akt) signaling for the downstream activation of IFN and pro-inflammatory cytokine-mediating genes. In vitro assays have demonstrated that CPX can trigger the release of type I IFN, enhancing the immunogenicity of tumor cells directly as well as improving anti-tumor immunity mediated by key immune cell types such as dendritic cells, CD4^+^ and CD8^+^ T cells, macrophages, and natural killer (NK) cells [25,26,27,28,29,30,31,32].

Type I IFN release has been shown to enhance dendritic cell activation and antigen presentation through the up-regulation of co-stimulatory molecules CD80/86 and CD40, as well as MHC class II expression in vivo. Type I IFN-stimulated dendritic cells display an improved capacity to prime both CD4^+^ and CD8^+^ T cell responses in multiple preclinical models of solid tumors. In a report published by Schiavoni et al. in 2011, low-dose CPX was shown to release significant amounts of type I IFN, leading to the superior induction of CD8α^+^ dendritic cells and priming of IFN-γ^+^ CD8^+^ T cells in an EG7 thymoma mouse model [33]. In a model of murine glioma, Du et al. (2020) demonstrated that low-dose CPX generated type I IFN release from GL261 and CT-2A murine glioma cells in vitro [34]. These results were then followed by in vivo studies in GL261 tumor-bearing mice, showing that tumor peptide vaccine synergizes with type I IFN release induced by CPX in vivo. These studies highlight the therapeutic potential of low-dose CPX to bolster the clinical efficacy of other novel immunotherapies against multiple tumor types.

## 7. CPX’s Influence on Th_1_ Immunity

Durable anti-tumor immunity is contingent on a multitude of concurrent immunological signaling cascades and activation of long-term effector immune cell activity. Broadly speaking, Th_1_ immune responses often take place in response to intracellular pathogens but are also crucial for anti-tumor immunity. Th_1_ immune responses are of a cell-mediated phenotype and are orchestrated by dendritic cells, CD8^+^ T cells, NK cells, M1 macrophages, and CD4^+^ T cells. Extensive study into how low-dose CPX can shift the immune phenotype post-treatment has indicated that low-dose CPX can restructure the TME to produce Th_1_ immunity. A study published by Malvicini et al. in 2009 showed that low-dose CPX in conjunction with IL-12 synergized to induce superior levels of CD4^+^ Th_1_ cells producing IFN-γ in an in vivo model of CT26 colon adenocarcinoma [35]. Likewise, a publication by Matar and colleagues in 2002 indicated that a single low-dose injection of CPX into lymphoma-bearing rats shifted splenic immune cell phenotypes from predominantly Th_2_ skewed to Th_1_ skewed, once again leading to superior production of IFN-γ and TNF-α-producing T cells [36]. The levels of immune suppressive cytokines IL-10, TGF-β, and nitric oxide were also highly reduced, further showing low-dose CPX’s ability to shift the immune response phenotype to an anti-tumor, cell-mediated Th_1_ phenotype.

## 8. Metronomic Low-Dose Administration of Chemotherapy

Canonically, chemotherapy is typically administered to cancer patients for the purpose of killing as many actively dividing and proliferating tumor cells as possible to maximize the patients’ chances of survival. Moreover, chemotherapy is generally given at or near the maximum tolerated dose, and therefore it often leads to severe toxicity and the potential for long-term complications in patients who are long-term survivors. In addition, while conventional high-dose CPX administration can directly kill tumor cells for tumor debulking, this high-dose administration often depletes key immune cell populations such as T cells, NK cells, and dendritic cells, which are crucial for robust anti-tumor immunity. High-dose administration of CPX is often associated with significant toxicities and side effects, including anemia, neutropenia, nausea, vomiting, cognitive fog, and infertility [37,38]. More severe side effects such as cardiac toxicities and hemorrhagic cystitis have also been documented during high-dose CPX treatment. These side effects often have significant adverse effects on the overall quality of life during and potentially after treatment. Over the past several years, the notion of low-dose metronomic chemotherapy administration has garnered more attention in preclinical cancer models as well as human clinical trials of various cancer types to reduce severe toxicities [39,40]. This treatment approach involves administering continuous, regularly scheduled, low doses of chemotherapy as a supplemental treatment to standard of care cancer immunotherapies. The advantages of low-dose metronomic chemotherapy are numerous, including unique immunomodulatory properties of chemotherapy on the TME as well as tumor cells individually, the decreased angiogenesis of solid tumors, and significantly less severe toxicity [37,38,39,40,41,42]. Evidence for CPX’s anti-angiogenic effects was published by Peyrl et al. (2023), who demonstrated that a dual treatment of low-dose oral tablet CPX plus etoposide in conjunction with bevacizumab exhibited potent anti-angiogenic properties in patients with recurrent medulloblastoma [41]. The median overall survival was 25.5 months with a progression-free survival (PFS) of 8.5 months. It was also noted that of the patients who exhibited a positive clinical response, the 5-year PFS was 66.7%, highlighting the potential anti-angiogenic properties of low-dose CPX and etoposide.

## 9. Metronomic Low-Dose Administration of CPX to Enhance Existing Cancer Immunotherapy in Preclinical Cancer Models

Due to the growing body of data highlighting the immunomodulatory effects of metronomic CPX, researchers have begun evaluating the efficacy of combining metronomic chemotherapy with current immunotherapies in both preclinical models and human clinical trials [11,42,43,44,45,46,47,48,49,50]. In this section, we will provide an overview of the increasing evidence for the therapeutic efficacy of metronomic CPX treatment in conjunction with existing cancer immunotherapies. Through testing with in vivo preclinical models of cancer, metronomic CPX has been shown to enhance the effectiveness of immune checkpoint inhibitors, tumor peptide vaccines, and other immunomodulatory anti-cancer agents [11,51,52,53,54]. For example, in a report published by Weir and colleagues in 2014, metronomic low-dose CPX was demonstrated to enhance anti-tumor immunity in conjunction with a novel tumor peptide vaccine that was tested in C3 tumor-bearing mice [52]. ELISpot and flow cytometry assays showed significant increases in IFN-γ-secreting CD8^+^ T cells. Adoptive transfer experiments indicated durable immunological memory that was transferable from mice treated with low-dose CPX and peptide vaccine to naïve mice. Park et al. published in 2011 that low-dose CPX plus dendritic cell vaccine derived from irradiated tumor cells improved the control of tumor growth kinetics, reduced intratumoral regulatory cells, and reduced levels of immune suppressive IL-10 and TGF-β in a model of mammary carcinoma [53]. Pfirschke et al. (2016) showed that metronomic low-dose CPX and oxaliplatin combined with either anti-CTLA4 or anti-PD-1 blockade generated superior induction of CD8^+^ T cells in a TLR-4 dependent manner in a model of pancreatic ductal adenocarcinoma [54].

## 10. Metronomic Low-Dose Administration of CPX to Enhance Existing Cancer Immunotherapy in Clinical Trials

The metronomic administration of chemotherapeutic agents such as CPX has become increasingly prevalent in clinical trials for solid tumor treatment [42,43,44,45,46,47,48,49,50]. Several examples of clinical investigations combining metronomic CPX treatment with immunotherapies are shown in Table 1. A Phase II clinical trial published by Mo and colleagues in 2024 showed superior clinical efficacy in patients with metastatic HER-2+ breast cancer treated with a dual treatment of low-dose CPX and toripalimab anti-PD-1 checkpoint blockade [42]. In a study published by Zsiros and colleagues in 2021, a Phase II clinical trial of recurrent ovarian cancer also showed that low-dose CPX and anti-PD-1 checkpoint blockade was well tolerated and 95% of patients experienced clinical benefit [43]. Interestingly, 25% of patients who participated in the trial observed a long-term treatment response of greater than 12 months, suggesting that metronomic chemotherapy and immune checkpoint blockade synergized positively in a manner that resembles the effects in preclinical studies.

## 11. Discussion

Chemotherapeutic agents such as CPX have been widely adopted in the standard of care treatment regimens for many solid and hematological malignancies. Despite their ability to directly kill cancer cells for tumor debulking, chemotherapies also often exhibit unacceptable toxicity. Given these clinical limitations, both clinicians and researchers have explored administering chemotherapies such as CPX in a metronomic dosing schedule, showing highly reduced toxicities and an objective clinical response, even in relapsed and refractory disease [55,56,57,58,59,60]. Although there are very limited human clinical data directly comparing metronomic CPX to conventional dose CPX, multiple human studies clearly show favorable toxicity and safety profiles using metronomic dosing. Studies directly comparing a metronomic or conventional dosage scheduling of CPX in preclinical models indicated that metronomic CPX was superior in reducing intratumoral Tregs in conjunction with increasing CD8^+^ T cell and NK cell activity [61,62,63,64,65]. Preclinical studies have also reported increased dendritic cell activation and antigen presentation through the induction of ICD post-metronomic CPX treatment, and multiple effects on the cytokine milieu and composition of the TME have also been documented in both preclinical and clinical studies of metronomic CPX [66,67,68,69]. This suggests that the use of metronomic CPX in conjunction with other immunotherapies such as immune checkpoint inhibitors could be used to achieve stable disease and objective clinical responses with substantial reductions in significant adverse events, facilitating a better quality of life during and after cancer treatment. Additional studies will be needed to completely elucidate the clinical efficacy of metronomic CPX as opposed to conventional dose CPX in the treatment of solid and hematological malignancies. Currently, there is a dearth of studies in the scientific literature that clearly delineate the circumstances and patient populations in which metronomic CPX treatment would be clinically optimal. This is compounded by the fact that the interrelated mechanisms of metronomic CPX’s effects on immune cells and tumor cells is still not fully elucidated. Further characterizing the effects of metronomic CPX in restructuring the immunopeptidome and activation of innate immune sensors of tumor cells is of considerable interest for future studies. This mechanistic insight could drive further development of tumor peptide vaccines and/or pairing of innate immune sensor agonist therapies with metronomic CPX. Additional studies will need to be conducted to stipulate clearly what dose of CPX is best suited for specific tumor and molecular subtypes on the metronomic dosing schedule, as well as to identify better therapeutic biomarkers for potential responses to metronomic CPX. Characterizing the importance of the frequency and dosage of metronomic CPX is also urgently needed to enhance the efficacy and tolerability of metronomic CPX. These novel avenues have the potential to enhance the immunogenicity of canonically cold tumor types and provide further optimized cancer treatment.

In addition to further characterizing the immunological mechanism of metronomic CPX, the usage of metronomic CPX in the context of palliative care is an emerging field of interest, particularly in geriatric and pediatric populations. While there is a paucity of research investigating the therapeutic efficacy of metronomic CPX in the context of palliative care, the limited literature on this topic has shown promise at slowing disease progression, while simultaneously increasing the quality of life during palliative care [70,71,72,73,74,75]. This approach of metronomic CPX would be highly attractive in patients who have limited ability to tolerate conventional therapeutic doses of chemotherapy due to pre-existing health conditions. Larger and more diverse tumor type clinical studies will need to be performed to elucidate these gaps in mechanism and identify optimal patient populations in both palliative and non-palliative settings.

## 12. Conclusions/Perspectives

In summary, the alkylating agent CPX has a well-documented set of novel immunomodulatory properties that have a strong translational application to the treatment of many solid and hematological malignancies. Moreover, the well-established direct impact of CPX on tumor cells complements the numerous effects of CPX on the TME that can serve to significantly alter the immune landscape of solid tumors that are often immunologically cold and unresponsive to immunotherapies (such as immune checkpoint blockade). This review also presents the applicability of the metronomic dosing schedule of CPX to synergize with other anti-cancer therapeutics while limiting overall toxicity and complications that are commonly seen in the canonical administration of chemotherapy agents. Further preclinical testing and human clinical studies are required to characterize the effects of CPX on the cancer TME when combined with other immunotherapies, to optimize the delivery strategies by which CPX can be administered, and to determine how it alters the immune suppressive nature of stromal cells and immune cell populations.

## Figures and Tables

**Table 1:** Clinical trials of metronomic (MN) CPX and immunotherapy combinations (from 2016 to 2025).

Phase and No. of Patients	Cancer	Chemotherapy	Immunotherapy/Target	Primary Outcome	Identifier No.	Reference
Phase II, 40 patients	Recurrent ovarian cancer	MN CPX	Pembrolizumab (anti-PD-1) and bevacizumab (anti-VEGF)	3 (7.5%) had CRs, 16 (40.0%) had PRs, and 19 (47.5%) had stable disease post-treatment, with an ORR of 47.5%, clinical benefit in 38 (95.0%), and durable response in 10 (25.0%). Median PFS was 10.0 months.	NCT02853318	[43]
Phase II, 35 patients	Metastatic castration-resistant prostate cancer	MN CPX	Personalized tumor peptide vaccine	Patients with positive immune responses showed significantly longer survival than those with negative responses, with median OS of 27.1 months and 15.4 months, respectively.	UMIN000005329 *	[45]
Phase II, 43 patients	Metastatic breast cancer	MN CPX, MN vinorelbine, MN capecitabine	Toripalimab (anti-PD-1)	The median PFS of patients in the VEX cohort was the longest, reaching 6.6 months, followed by the bevacizumab (4.0 months) and cisplatin (3.5 months) cohorts.	NCT04389073	[42]
Phase I, 21 patients	Metastatic melanoma	MN CPX	Autologous dendritic cell vaccine	Well tolerated and favorable safety profile.	NCT00978913	[46]
Phase II, 13 patients	Desmoplastic small round cell tumor, neuroblastoma, and high-grade glioma	MN CPX	Nivolumab (anti-PD-1)	Well tolerated and favorable safety profile but limited clinical benefit.	NCT02813135	[47]
Phase I, 16 patients	Relapsed and refractory pediatric solid tumors	MN CPX, MN capecitabine and vinblastine	Nivolumab (anti-PD-1)	Well tolerated and low toxicity.	NCT03585465	[48]
Phase II, 20 patients	Head and neck squamous cell carcinoma	MN CPX and radiation	Avelumab (anti-PD-L1)	Favorable tolerability but limited clinical benefit.	EudraCT 201700035339 *	[49]
Phase II, 20 patients	Soft tissue sarcoma	MN CPX	Oncolytic virus	Well tolerated and low toxicity.	NCT02630368	[50]

* Denotes trial was conducted outside of the U.S. U.S. clinical trials can be found at ClinicalTrials.gov. Abbreviations used in the table: CR, complete response; CPX, cyclophosphamide; MN, metronomic; ORR, overall response rate; OS, overall survival; PD-1, programmed cell death protein 1; PD-L1, programmed death-ligand 1; PFS, progression-free survival; VEGF, vascular endothelial growth factor; VEX, regimen of metronomic cyclophosphamide, vinorelbine, and capecitabine.

## Abbreviations

The following abbreviations are used in this manuscript:

ATP	adenosine triphosphate
CR	complete response
CRT	calreticulin
CPX	cyclophosphamide
DNA	deoxyribonucleic acid
HMGB1	high-mobility group box protein 1
ICD	immunogenic cell death
IFN	interferon
IL	interleukin
JAK/STAT	Janus kinase/signal transducers and activators of transcription
MAPK	mitogen-activated protein kinase
MDSC	myeloid-derived suppressor cell
MHC class I	major histocompatibility complex class I
MHC class II	major histocompatibility complex class II
NF-κB	nuclear factor kappa B
NK cell	natural killer cell
ORR	overall response rate
OS	overall survival
PFS	progression-free survival
PD-1	programmed cell death protein 1
PD-L1	programmed death-ligand 1
PI3K/Akt	phosphoinositide 3-kinase/Akt
TGF-β	transforming growth factor beta
Th_1_	T helper 1
TME	tumor microenvironment
TNF-α	tumor necrosis factor alpha
Treg	regulatory T cell
VEGF	vascular endothelial growth factor
VEX	treatment regimen of metronomic cyclophosphamide, vinorelbine, and capecitabine
